# The Usefulness of Genotyping of Celiac Disease-Specific HLA among Children with Type 1 Diabetes in Various Clinical Situations

**DOI:** 10.1155/2020/7869350

**Published:** 2020-02-22

**Authors:** Grazyna Deja, Dominika Sikora, Aleksandra Pyziak-Skupien, Karolina Klenczar, Rafał Deja, Przemysława Jarosz-Chobot

**Affiliations:** ^1^Department of Children's Diabetology, Medical University of Silesia in Katowice, Poland; ^2^Students' Scientific Association in Department of Children's Diabetology, Medical University of Silesia, Katowice, Poland; ^3^Department of Computer Science, WSB University, Dabrowa Gornicza, Poland

## Abstract

**Aim:**

The aim of the study was to determine the usefulness of HLA DQ2/DQ8 genotyping in children with T1D in various clinical situations: as a screening test at the diabetes onset, as a verification of the diagnosis in doubtful situations, and as a test estimating the risk of CD in the future. *Materials and methods*. Three groups of patients with T1D were included: newly diagnosed (*n* = 92), with CD and villous atrophy (*n* = 30), and with potential CD (*n* = 23). Genetic tests were performed (commercial test, PCR, and REX), and clinical data were collected.

**Results:**

The results of genetic tests confirmed the presence of DQ2/DQ8 in 94% of children with diabetes (group I) and in 100% of children with diabetes and CD (groups II and III, respectively). Comparative analysis of the HLA DQ2/DQ8 distribution did not show any differences. Allele DRB1^∗^04 (linked with HLA DQ8) was significantly less common in children with diabetes and CD (group I versus groups II and III, 56.5% vs. 24.5%; *p* = 0.001). The probability of developing CD in DRB1^∗^04-positive patients was 4 times lower (OR 0.25; 95% CI 0.118-0.529; *p* = 0.001). DRB1^∗^04 was significantly less frequent in children with villous atrophy compared to potential CD (13% vs. 39%; *p* = 0.03).

**Conclusions:**

Genotyping HLA DQ2/DQ8 as a negative screening has limited use in assessing the risk of CD at the diabetes onset and does not allow to verify the diagnosis of CD in doubtful situations. The presence of the DRB1^∗^04 allele modulates the risk of CD and significantly reduces it and can predict a potential form.

## 1. Introduction

Celiac disease (CD) is defined as a systemic disease presenting with various symptoms or symptomless caused by gluten sensitivity, which is revealed in genetically predisposed people. The risk of development is 1% in the Caucasian race, with most cases being subclinical [1–4]. The main risk factor for CD is genetic predisposition: the presence of typical HLA DQ2 (90-95%) and DQ8 (5-10%) haplotypes. Therefore, it is hypothesized that the presence of these haplotypes does not determine the development of the disease, but their absence excluded CD even in 100%. Since 2012, the recommendations of ESPGHAN (European Society of Pediatric Gastroenterology, Hepatology and Nutrition) have changed the diagnostic algorithm for CD by including the HLA typing [1]. Two diagnostic algorithms have been developed: for people with symptoms of CD and for people at risk, including patients with type 1 diabetes (T1D). HLA genotyping provides additional information for commonly used serological tests. It can, therefore, be used as a tool for negative screening in risk groups or to confirm a diagnosis in doubtful, subclinical cases, which represent the majority. The coincidence of CD with T1D for different populations is widely known—according to various sources, it oscillates around 6-15% [4–8], albeit it is very problematic to make a proper diagnosis in asymptomatic patients, mostly due to inconclusive results of small intestine biopsies [3, 4, 9] and the phenomenon of negative seroconversion [10] (decrease in tTG antibody levels, despite the lack of a gluten-free diet).

The currently recommended diagnostic scheme of CD in groups of risk with HLA typing is staged mostly in order to rule out the disease, so patients with T1D would not have to perform annually immunological tests. However, due to the high (above 90%) similarity of haplotypes HLA-DQ2/DQ8 in T1D and CD, the real usefulness of HLA typing, as a first-line screening tests, in children with T1D is relatively low, as confirmed by studies conducted in various populations [5, 6, 11, 12]. On the other hand, studies assessing the usefulness of haplotyping in doubtful situations, so common in everyday diabetological practice, e.g., with inconclusive serological and histopathological results in patients without clinical symptoms of disease, or in patients on a gluten-free diet, but without confirmation of villous atrophy, are uncommon.

The aim of our study was to determine the usefulness of genetic tests specific for CD—haplotypes HLA DQ2/DQ8—among children with T1D in various clinical situations: as a screening test at the diabetes onset and verification of the diagnosis of CD in doubtful situations. Furthermore, the relation between the said haplotypes and clinical factors is going to be determined, in the context of CD appearance and its clinical picture, in order to, based on all analyzed parameters, more precisely assessing the risk of onset CD in the future.

## 2. Materials and Methods

### 2.1. Patients

The study was conducted in regional reference diabetological center, Department of Children's Diabetology of the Medical University of Silesia in Katowice, Poland, among children with T1D. Three groups of patients were included in the study. The group I was composed of children who underwent screening for CD at the onset of T1D. Additionally, children with diabetes and various forms of CD diagnosed in the past, remaining under the care of the outpatient's diabetes clinic, were assigned to the study: children with CD with villous atrophy were included in group II and children with potential CD, in group III.

All consecutive patients with T1D onset confirmed by immunological tests hospitalized in the Dept. of Children's Diabetology were prospectively collected for the period of 6 months (in 2016) and included in group I (92 children). Screening for CD was routinely performed on the basis of standard immunological tests (total IgA, and IgA tTG, in case of IgA deficiency in IgG class) and genetic tests (haplotypes predisposing to CD).

Children, having CD diagnosed earlier, randomly selected from those remaining under the care of the outpatient's diabetes clinic, were also included to the study. According to ESPGHAN, ISPAD, and Polish Diabetes Recommendations [1, 3, 12], all patients in our center with symptoms suggestive of CD and/or elevated immune markers underwent small intestine biopsy. CD was diagnosed based on Marsh criteria. In case of villous atrophy (grade 3), gluten-free diet was introduced. Potential CD was diagnosed in patients without villous atrophy. In this group, monitoring of antibodies and clinical symptoms was recommended, but gluten-free diet was not introduced. For the purpose of the analysis, children with CD with villous atrophy were included in group II (30 children) and children with potential CD, in group III (23 children). In all children with T1D and CD recruited for the study, genetic tests were performed and clinical data were collected, including sex, age at T1D onset, results of immunological tests confirming the diagnosis of T1D, age of CD onset, and results of tTG tests at the diagnosis. The study was approved by the local bioethics committee, and all genetic tests were performed after having obtained the informed consent from parents/children.

### 2.2. Laboratory Tests

The presence of haplotypes associated with predisposition to CD was evaluated in all patients. Analysis of haplotypes HLA-DQ2 (DQA1^∗^05, DQB1^∗^02), HLA-DQ8 (DQA1^∗^03, DQB1^∗^0302), and DRB1^∗^04 allele (linked with HLA-DQ8) was performed. Diagnostic material was a cheek swab, where genomic DNA from epithelial cells was acquired. Genotyping was performed using commercial tests: the Real-Time PCR (REX Company S-A).

In all groups, retrospective data of the level of total IgA, IgA tTG, and, in case of IgA deficiency, IgG tTG, or IgG EmA, and ZnT8, IA2, and GAD IgA, was collected. IgA tTG and IgG tTG (cut-off 20 RU/ml) were analyzed in the blood serum by ELISA methods. GAD, IA2, and ZnT8 antibody levels were evaluated by immunoenzymatic method (ELISA RSR GADAb, RSR, UK; ELISA RSR IA2Ab, RSR, UK; and ELISA RSR ZnT8Ab, RSR, UK, respectively). The reference values were as follows: for GAD antibodies, <10 U/ml; for IA2 antibodies, <10 U/ml; and for ZnT8 antibodies <15 U/ml.

### 2.3. Statistical Analysis

Categorical variables were presented as numbers with appropriate percentages and continuous variables, as medians with interquartile range (25%-75%). Verification of the normality of distribution was carried out using the Shapiro-Wilk test.

To compare the age of diagnosis and the antibody concentration in the studied groups, Mann–Whitney test was performed. Receiver Operating Characteristic (ROC) curves for celiac occurrence model with the calculation of the area under the ROC curve (AUC) were evaluated. 95% Confidence Intervals (95% CI) were computed for AUCs. The strength of the combined effect of these parameters was determined by the odds ratio (OR) with confidence limits (+95% CI; -95% CI). Results with *p* values < 0.05 were considered as statistically significant. Analyses were performed using Statistica 13.1 PL software (Statsoft, Tulsa, OK, USA).

## 3. Results

Characteristics of the studied groups and the results of genetic tests are presented in Table 1. As a result of immunological screening for CD in group I, elevated tTG antibody titer was found in 2 patients. After small intestine biopsy, CD with villous atrophy was diagnosed in one child and potential CD, in the second one. These children were included in the statistical analysis to the appropriate groups.

**Figure 1:**
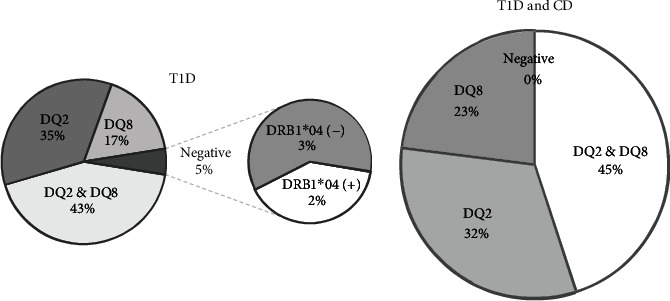
Distribution of HLA haplotypes in group I (T1D) and groups II and III (T1D and CD); *p* = 0.321.

The lack of a genetic predisposition to developing CD at the onset of T1D (group I), i.e., the absence of HLA DQ2/DQ8, was observed in 5% of children, of which 2% were found only DRB1^∗^04 allele (Figure 1). The presence of typical haplotypes predisposing to CD was confirmed in all children with T1D and CD (groups II and III) (Figure 1). Comparative analysis of the distribution of haplotypes in all of the groups did not show any differences in HLA DQ2/DQ8 occurrence, but it was observed that allele DRB1^∗^04 (linked with HLA DQ8) was significantly less common in children with T1D and CD (group I versus groups II and III) (57% vs. 25%; *p* = 0.001) (Table 1). The probability of developing CD in patients with the DRB1^∗^04 allele in studied cohort was four times lower (OR 0.25; 95% CI 0.118-0.529; *p* = 0.001). Comparison of groups II and III (different forms of CD) did not show significant differences in the distribution of haplotype DQ2; however, the DRB1^∗^04 allele was found less frequent in the group of children with CD with villous atrophy (13% vs. 39%; *p* = 0.305) (Table 1).

The relationship between the presence of individual haplotypes DQ2/DQ8 and the immune response (presence of antibodies) was found (Table 2). In addition, the presence of DRB1^∗^04 allele was significantly associated with an older age of diabetes diagnosis, lower tTG titer, lower GAD, and higher IA2 and ZnT8 titers, as presented in Table 3.

**Figure 2:**
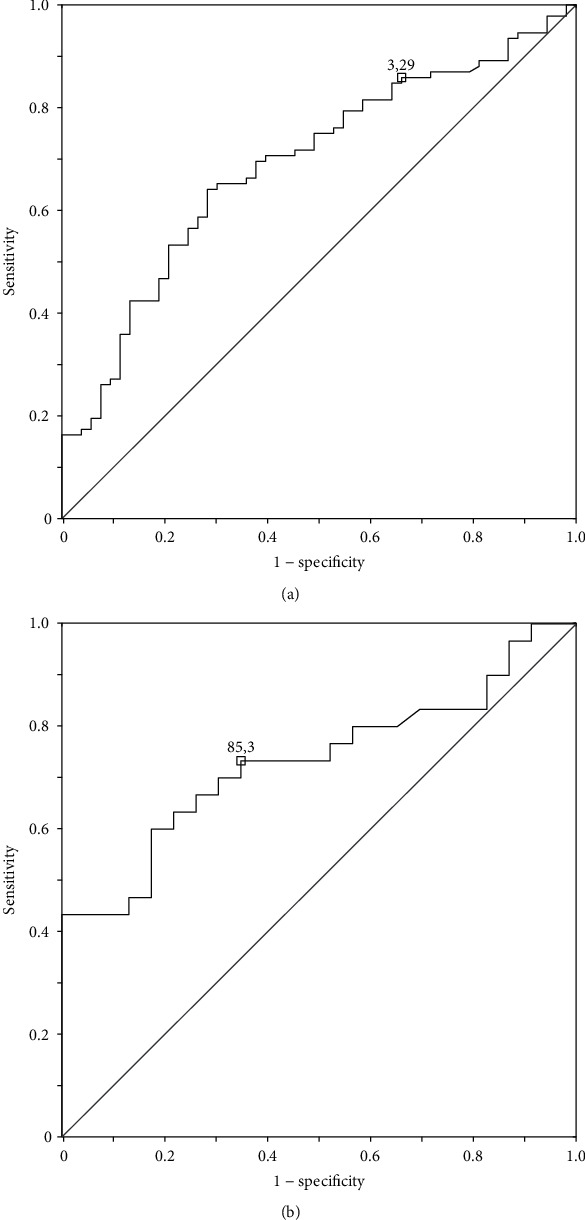
(a) ROC curve for age of diabetes diagnosis predisposing for CD (AUC 0.689 (95% CI 0.602, 0.776); *p* = 0.001). (b) ROC curve for TTG antibody titers predisposing for CD with villous atrophy (AUC 0.744 (95% CI 0.613, 0.876); *p* = 0.001).

Evaluating the clinical parameters in the context of the risk of developing CD, it was found that the age of T1D onset in children with coexisting T1D and CD was significantly lower compared to the age of children with T1D alone (groups II and III vs. group I) (Me 5.42 (25-75%, 2.84 – 8.71) vs. Me 9.99 (25-75%, 5.32-13.52); *p* = 0.001) (Table 1). The optimal cut-off point in ROC analysis for age of diabetes diagnosis for patients with coexisting CD was 3.29 years (85.9% sensitivity, 34.0% specificity, AUC 0.689 (95% CI 0.602, 0.776); *p* = 0.001) (Figure 2(a)). The probability of developing CD in patients with T1D onset below 3.5 years of age was three times higher (OR 2.868; 95% CI 1.304-6.309; *p* = 0.008) in our cohort.

Children with various forms of CD did not differ significantly in the age of diabetes onset and immunological markers associated with the diagnosis of T1D (Table 1). Significantly higher tTG antibody titers were found in group II vs. group III (Me 160 (25-75%, 72-704) vs. Me 68 (25-75%, 50-119); *p* = 0.003). Based on the ROC curve, the optimal cut-off point of tTG antibody titer for CD with villous atrophy was 85.3 units with 73.3% sensitivity and 65.2% specificity (AUC 0.744 (95% CI 0.613-0.876); *p* = 0.001) (Figure 2(b)).

## 4. Discussion

High prevalence of CD among diabetic patients and huge influence on clinical course of diabetes, often asymptomatic process and diagnostic difficulties, make the problem of correct diagnosis of CD in risk group still valid and not yet fully solved. Modification in 2012, by ESPHGAN, the long-standing rules of CD screening, among diabetic patients, through the introduction of HLA genotyping unfortunately has not fulfilled the hopes for simplification of this procedure. Our study was the first to evaluate the Polish population in the context of negative screening for CD in children with T1D. We confirmed the low usefulness of HLA genotyping, where only 5% of all the prospectively collected children with diabetes onset, included to the study within 6 months, had a negative result.

In the majority of patients, the presence of typical, for both diseases, haplotypes HLA-DQ2 (DQA1^∗^05, DQB1^∗^02) and HLA-DQ8 (DQA1^∗^03, DQB1^∗^0302) or both was confirmed (in 34%, 19%, and 44%, respectively). The literature data show that, depending on the population, negative screening rates are different and for most European countries remain at a very low level—a few/twelve percent [2, 6, 11, 13, 14]. Moreover, the low usefulness of HLA genotyping in another contexts has been recently described in a study based on the data from the international DPV registry (Diabetes Prospective Follow-up; including >75000 patients with T1D, with HLA testing in >1600 children) [15]. The authors analyzed the history of CD diagnoses and observed that even in HLA-negative patients, both positive markers of CD (49 out of 234 patients) and the CD itself confirmed with biopsy (7/234) were relatively frequent. Strictly following ESPGHAN recommendations, i.e., performing HLA as a negative first-line screening that excludes the need for further observation, would not be the right procedure for such patients. Furthermore, analyses of the costs of performing HLA genotyping, as a preliminary risk verification, showed that such proceeding is not justified [6, 15]. Therefore, the latest ISPAD 2018 recommendations conclude that genotyping is not a currently recommended method of screening for CD in patients with T1D [3].

The general distribution of DQ2/DQ8 haplotypes between our study groups did not differ significantly. However, we observed differences in the distribution of DRB1^∗^04 allele; it was significantly less frequent in the group of children with diabetes and coexisting CD, modulating the risk of CD occurrence (OR = 4).

It is known from the literature that the DRB1^∗^04 allele is strongly associated with the predisposition to many autoimmune diseases T1D, autoimmune Addison's disease [16], multiple sclerosis [17], and rheumatoid arthritis [18], but the CD is not mentioned among them. Moreover, the presence of this allele correlates with the later onset of diabetes and the specific immune response higher ZnT8 and IA2 titer and lower GAD titer, which was described earlier in another studies, and now, we also confirmed these correlations [9, 19].

The occurrence of CD is inversely and independently related to the age at the time of the diabetes onset, with the highest risk in people with diabetes diagnosed before the age of 5 [20–22]. This trend was also observed in our study—the age at onset of diabetes in children who developed CD was significantly lower than the average age at onset of T1D in the cross-sectional population. Moreover, in our study, the cut-off point determined on the baseline ROC curve was <3.5 years, which is considerably less than the limit of 5 years suggested above. This decrease in age limit may be a phenomenon parallel to the general trend observed for the Polish population in the last two decades of a significant decrease of age at T1D onset [23]. This probably indicates a strong influence of the same local environmental factors inducing autoaggressive processes at a very early age, when the immune system is not yet fully mature. Our results should be treated as an outline, due to a small quantity of the group, showing a distinct trend and phenomenon typical to countries of our region, suggesting the necessity of particularly thorough observation of the youngest children for CD.

Another aim of our study was to evaluate the usefulness of HLA genotyping as an additional test in doubtful situations, e.g., to differentiate between children with villous atrophy and potential CD. A general comparison of the distribution of HLA DQ2/DQ8 haplotypes did not show differences between groups, except for the tendency to reduce the risk of CD with villous atrophy in DRB1^∗^04 allele-positive patients. Our observations are in accordance with the works of Tucci et al. [24] and Auricchio et al. [25], who, in addition to the classical HLA testing, analyzed the distribution of several polymorphisms of single “candidate genes” related to the pathogenesis of potential CD. They confirmed that children with potential CD have a slightly different genetic background—the disease is less frequently associated with high-risk HLA genes and connected with other gene polymorphisms, which determine the course of immune reactions occurring in the intestinal mucosa, what makes the immune response less pronounced. Therefore, the role of HLA in diagnosing celiac disease in doubtful situations seems to diminish significantly. Auricchio [25] showed in his study that in the model, assessing the risk of developing atrophic villi in 9-year observation of patients with potential CD, only genetic factors not related to HLA, like SNP in IL12A, OLIG3/TNFAIP3, and IL2/IL21, were significantly connected with the risk of classical CD. The suggestions that non-HLA genes and environmental factors play also a significant role in determining the phenotype of CD were recently published by Kauma et al. [26] in a study comparing clinical outcomes in pairs of affected siblings. The phenotype was categorized into gastrointestinal, extraintestinal, malabsorption/anemia, and asymptomatic, and the pairs with discordant presentation had similar HLA haplotypes more often than the concordant pairs. It is possible that a similar phenomenon will be found in CD with and without villous atrophy. CD especially in risk groups presents itself in a milder form. Current ISPAD recommendations suggest that biopsy should be taken from, at least, several locations because of nonfocal or “patchy” histopathologic lesions that have been observed from samples in over 50% of patients [3, 27, 28]. Moreover, the phenomenon of negative seroconversion or long-term fluctuations in the level of antibodies, with not very high titers, is common [10, 25]. In our study, mean tTG concentrations in the group of patients with potential CD were significantly lower, compared to the form with villous atrophy (73.6 IU/ml vs. 160 IU/ml). The cut-off point, determined on the basis of the ROC curve, was the value of tTG 85 IU/ml, which is four times higher than the laboratory standard. This value is much lower than the threshold of 10-fold exceeding the norm proposed in ESPGHAN recommendations, which determines the possibility of abandoning the classical small intestine biopsy—such tTG value was presented only in 3 (10%) of our patients with CD with villous atrophy. Although explanation of our observation could result from the fact that most of our cases were asymptomatic, and actively screened, the proper diagnosis was made relatively early. But once again, clinical practice indicates that ESPGHAN recommendations are of limited use in the diagnosis process of CD in children with T1D.

The limitation of our study is a small size of patients included, but having taken into account that Polish population is fairly homogenic when talking about nationalities, which means it is also quite stable in the distribution of haplotypes predisposing to getting CD or T1D, it ought not to have any significant impact on the results of our study.

All in all, our study, being the first in Polish population, assessing the usefulness of celiac HLA genotyping screening in children with T1D, in general, shows a small importance in practice because of the wide distribution of HLA DQ2/DQ8 haplotypes in our cohort. However, we have found that the usage of commercial, widely available, relatively cheap genetic tests allows stratifying the risk of developing CD. Regardless of the DQ2/DQ8 haplotypes, having the DRB1^∗^04 allele in our cohort reduced the risk of CD in general, as well as CD with villous atrophy, which may have some significance in the context of further screening in suspected patients. But taking into account the abundance of publications pointing out the lack of practicability, lack of cost-effectiveness, and potential psychological burden in an unclear situation, one should agree with the new ISPAD recommendations that do not recommend HLA genotyping as a screening method. So, is it time to change the general recommendations formulated by ESPGHAN in diagnostic process of CD in risk groups?

## Figures and Tables

**Table 1:** Characteristic of the studied groups and the results of the genetic tests.

	Group I: 92 (63%)	Group II: 30 (21%)	Group III: 23 (16%)	Groups II and III: 53 (37%)	*p* value group I (T1D) vs. groups II and III (CD)	*p* value group II vs. group III	*p* value (all groups)
Female (*n* (%))	32 (35%)	16 (53%)	8 (35%)	24 (45%)	NS	NS	NS
DQA1^∗^05 (*n* (%))	56 (61%)	21 (70%)	15 (65%)	36 (68%)	NS	NS	NS
DQB1^∗^02 (*n* (%))	65 (71%)	24 (80%)	16 (70%)	40 (75%)	NS	NS	NS
DQA1^∗^03 (*n* (%))	15 (16%)	4 (13%)	3 (13%)	7 (13%)	NS	NS	NS
DQB1^∗^0302 (*n* (%))	53 (58%)	18 (60%)	17 (74%)	35 (66%)	NS	NS	NS
DRB1^∗^04 (*n* (%))	52 (57%)	4 (13%)	9 (39%)	13 (25%)	0.001	0.031	0.001
Age at T1D diagnosis (years) (Me (IQR))	9.99 (5.32–13.52)	5.19 (2.38–9.10)	5.60 (3.16–7.88)	5.41 (2.84–8.71)	0.001	NS	0.001
Age at CD diagnosis (years) (Me (IQR))		7.30 (5.10–11.10)	6.96 (4.80–11.53)	7.23 (5.04–11.32)	—	NS	NS
TTG (U/ml)	4.8 (3.3–7.6)	160.0 (71.9–704.0)	73.6 (51.0–118.0)	109.6 (60.0–321.0)	0.001	0.004	0.001
ZnT8 (U/ml)	221.0 (29.8–539.9)	110.0 (4.9–529.0)	166.3 (15.3–310.7)	120.0 (5.1–507.4)	NS	NS	NS
IA2 (U/ml)	216.0 (3.9–722.0)	21.7 (7.0–922.0)	185.9 (1.6–666.1)	96.6 (3.8–801.1)	NS	NS	NS
GAD (U/ml)	83.0 (6.7–560.0)	51.9 (5–375.0)	42.7 (13.3–402.6)	49.0 (8.6–395.2)	NS	NS	NS

**Table 2:** The association of haplotypes with immunological status (ZnT8, IA2, and GAD titers)—all groups.

	DQ2	DQ8	DQ2 and DQ8	Negative	*p* value (all groups)
ZnT8 (U/ml)	106.22 (14.29-509.52)	169.41 (10.11-623.88)	272.14 (18.77-529.48)	205.28 (81.95-678.8)	0.334
IA2 (U/ml)	44.29 (1.94-435.72)	543.07 (51.01-1047.4)	194.15 (5.145-646.77)	102.99 (4.1-501.61)	0.018
GAD (U/ml)	222.84 (16.86-1139.94)	10.52 (2.9-56)	76.105 (9.705-435.79)	151.29 (6.7-1509.65)	0.011

**Table 3:** The association of DRB1^∗^04 allele with clinical course of both diseases—all groups.

	Absent DRB1^∗^04	Present DRB1^∗^04	*p* value (all groups)
Age at CD diagnosis (years)	7.50 (5.51-11.28)	6.60 (4.69-11.53)	0.404
Age at T1D diagnosis (years)	6.22 (3.22-11.67)	8.52 (4.99-13.54)	0.041
TTG (U/ml)	21.90 (4.35-134.35)	6.40 (4.20-11.10)	0.008
ZnT8 (U/ml)	106.22 (13.77-457.79)	308.98 (45.41-554.13)	0.066
IA2 (U/ml)	23.46 (2.89-511.00)	393.16 (25.83-794.09)	0.009
GAD (U/ml)	151.72 (13.07-633.08)	21.83 (7.78-169.93)	0.043

## Data Availability

Data supporting the results could be added in the request of the reviewer.
